# Monitoring genotoxic, biochemical and morphotoxic potential of penoxsulam and the protective role of European blueberry (*Vaccinium myrtillus* L.) extract

**DOI:** 10.1038/s41598-023-34068-0

**Published:** 2023-04-26

**Authors:** Ali Acar, Divya Singh

**Affiliations:** 1 Department of Medical Services and Techniques, Vocational School of Health Services, Giresun University, Giresun, Turkey; 2 Central Sericultural Research and Training Institute, Mysore, India

**Keywords:** Plant stress responses, Plant sciences, Environmental sciences, Environmental chemistry

## Abstract

The present study aimed at exploring to explore the penoxsulam toxicity and protective effects of blueberry extract in roots of *Allium cepa* L. The effective concentration (EC_50_) of penoxsulam was determined at 20 µg/L by the root growth inhibition test as the concentration reducing the root length by 50%. The bulbs of *A. cepa* L. were treated with tap water, blueberry extracts (25 and 50 mg/L), penoxsulam (20 µg/L) and combination of blueberry extracts (25 and 50 mg/L) with penoxsulam (20 µg/L) for 96 h. The results revealed that penoxsulam exposure inhibited cell division, rooting percentage, growth rate, root length and weight gain in the roots of *A. cepa* L. In addition, it induced chromosomal anomalies such as sticky chromosome, fragment, unequal distribution of chromatin, bridge, vagrant chromosome and c-mitosis and DNA strand breaks. Further, penoxsulam treatment enhanced malondialdehyde content and SOD, CAT and GR antioxidant enzyme activities. Molecular docking results supported the up-regulation of antioxidant enzyme SOD, CAT and GR. Against all these toxicity, blueberry extracts reduced penoxsulam toxicity in a concentration-dependent manner. The highest amount of recovery for cytological, morphological and oxidative stress parameters was observed when using blueberry extract at a concentration of 50 mg/L. In addition, blueberry extracts application showed a positive correlation with weight gain, root length, mitotic index and rooting percentage whereas a negative correlation with micronucleus formation, DNA damage, chromosomal aberrations, antioxidant enzymes activities and lipid peroxidation indicating its protecting effects. As a result, it has been seen that the blueberry extract can tolerate all these toxic effects of penoxsulam depending on the concentration, and it has been understood that it is a good protective natural product against such chemical exposures.

## Introduction

In recent decades, the application of pesticides has been expanded throughout the world. Environmental pollution induced by the extensive use of pesticides has become a major concern in agriculture. Among pesticides, herbicides are applied to control weeds. The sulfonamide class of herbicides is employed on various crops to control grassy and broad-leaved weeds^1^. Several studies indicated the environmentally hazardous effects of sulfonamides in soil^2–4^. Pastures and legume crops are reported to be highly sensitive to sulfonamide residues in the soil even at trace levels^1^. Sulfonamide herbicides applied in agricultural fields may contaminate ground and surface water resources due to leaching and runoff processes. Their residues have been detected in the ground and surface water resources^2^.

Penoxsulam [2-(2,2-difluoroethoxy)-N-(5,8-dimethoxy-[1,2,4]triazolo[1,5-c]pyrimidin-2-yl)-6-(trifluoromethyl)benzenesulfonamide] is an active ingredient belongs to triazolopyrimidine sulphonamide class of herbicide class with the chemical formula C_16_H_14_F_5_N_5_O_5_S. It is absorbed by roots, leaves and shoots and thus translocated in plants^5^. Penoxsulam is a non-volatile, hydrolytically stable, water-soluble herbicide. It exhibits high herbicidal activity at low concentrations and in this way provides exceptional crop safety. Penoxsulam has exceptional herbicidal activity against numerous annual and perennial weeds particularly sedges and broad-leaved weeds^6^. It controls weeds by inhibiting acetolactate synthetase activity that is essential for the biosynthesis of branched-chain amino acids leucine, isoleucine and valine^7^. These amino acids are required for protein synthesis and, thus, necessary for cell metabolism^8^. The half-life of penoxsulam is varying from 3.10 to 27.70 days. However, the presence of penoxsulam in water and soil even in minute quantity possesses a potential risk to aquatic life, terrestrial plants and soil microbes^9,10^. Since 2008 in 28 countries, penoxsulam has been registered to control sedge, broadleaf weed and grass in water-seeded, direct-seeded and transplanted rice^11,12^. Nohatto et al*.*^13^ observed the penoxsulam-induced oxidative stress and alteration of antioxidant enzymes in rice plants. Penoxsulam might produce reactive oxygen species (ROS), lysosomal abnormalities and DNA damage in marine mussels^14^. Penoxsulam also causes oxidative stress in the muscles, brain and liver of silver catfish and carp^3,15^.

An imbalance in the production of ROS and antioxidant defense results in oxidative stress^16^. The antioxidant defense system consists of enzymatic and non-enzymatic antioxidants. The toxic compounds alter the activity of enzymatic antioxidants such as superoxide dismutase (SOD), catalase (CAT) and glutathione reductase (GR). The interaction of proteins with chemical compounds using a molecular docking software determine the most important interaction mode and protein-legend binding efficacy providing a 3D-crystal structure of the complexes. The excess ROS interacts with biomolecules leading to the inactivation of enzymes as well as DNA and RNA damage. ROS may cause lipid peroxidation that changes the structure and function of the cell membrane. Lipid peroxidation is a complex process resulting from the reaction of free radicals with unsaturated fatty acids^17,18^.

Vegetables and fruits are natural sources of antioxidants. Blueberry is a perennial plant that belongs to the family Ericaceae. Blueberry contains phenolic compounds (anthocyanins, catechins, quercetin, tannins, phenolic acids and ellagitannins), pectins, sugars and vitamins^19^. Anthocyanins are well-known antioxidants and also inhibit the oxidation of low-density proteins. They play an important role in the scavenging of free radicals, attenuation of oxidative stress, inhibition of NO production and improvement in antioxidant defenses^20,21^. Anthocyanins could play important role in the protection of DNA from the damage caused by peroxyl radicals^22^. The studies suggested that anthocyanins may intercalate with DNA forming a DNA co-pigment complex that involves in the regulation of gene expression and also protects DNA from oxidative damage. In vitro studies reported the anti-inflammatory, anti-apoptotic and anti-bacterial activity of anthocyanins^23–25^.

*Allium cepa* L. is an effective plant model for assessing the hazardous effects of environmental pollutants^26–29^. About 148 compounds have been evaluated by 164 *Allium* test and have shown 76% result similarities with other studies^30^. Therefore, *Allium* test has been accepted as a standard test for the identification of toxic chemicals^30^. *Allium cepa* L. is a very effective test model to study the cytotoxicity, genotoxicity and mutagenicity of toxic chemical compounds^31,32^. The similarity in toxicity test results of in vitro cell culture and in vivo animal studies has revealed the accuracy of the *Allium* test^33–36^. This test has been used to investigate the harmful effects of toxic substances on cell division and DNA damage^37^.

The absence of a comprehensive study that investigates the toxic effects of penoxsulam with multiple parameters, and the interrelationship between these parameters, as well as the scarcity of research on the protective effect of blueberry extract has led to the conception of investigation of both effects in this study. In this study, the effects of penoxsulam on cell division, root development and antioxidant enzymes along with its interactive potential were investigated on *Allium cepa* L. In addition, the therapeutic role of blueberry extracts was also assessed against these effects.

## Material and methods

The experiments based on plant were performed in accordance with international guidelines and legislation^38,39^. The similar size (25–35 mm diameter) bulbs of *Allium cepa* L. were procured from a local market. *A. cepa* L. (*Amaryllidaceae*) (2n = 16) were defined using taxonomic characters and approved at the Department of Botany, Faculty of Arts and Sciences, Giresun University. These bulbs were stored in a dry and cool place. Before treatment, bulbs were washed and brownish base plates along with external scales were removed.

### Determination of EC_50_ concentration

The *Allium* root growth test was used to determine the effective concentration of penoxsulam. The concentration of penoxsulam required to inhibit root length by 50% in comparison to the control was established as a half-maximal effective concentration (EC_50_). The bulbs were treated with penoxsulam in a range of 5–50 µg/L for 96 h at room temperature. Treatment of bulbs with distilled water was considered as a control. The solutions were changed after 24 h. An average of 50 roots from 6 bulbs was recorded to calculate EC_50_.

### Test material and treatments

The experiments were designed in six groups. The European blueberry extract (BBE) used in the test was purchased commercially (GNC, 60 mg in capsule) and the BBE content consists entirely of blueberry and does not contain any additives. Penoxsulam (C_16_H_14_F_5_N_5_O_5_S) is provided as a commercially available pesticide (25.2 g/L penoxsulam). It has been declared by the manufacturer that there is no additive other than penoxsulam in the pesticide content. The bulbs were treated with tap water in the control group whereas application groups were exposed to penoxsulam (20 μg/L), blueberry extracts (25 and 50 mg/L) and combination of penoxsulam and blueberry extract (Table 1) for 96 h at 24 °C. The application was carried out by keeping the base plates of the ampoules in contact with the solution. This was achieved by leaving the roots in contact with the solution. To prevent any changes in concentration, the solutions in contact with the bulbs were refreshed daily. About 10 bulbs from each group were selected for cytological and biochemical observations and 50 bulbs were chosen for physiological studies.

**Table 1:** Treatment groups.

Groups	Treatment
Group I	Tap water
Group II	25 mg/L BBE
Group III	50 mg/L BBE
Group IV	20 µg/L Penoxsulam
Group V	20 μg/L Penoxsulam + 25 mg/L BBE
Group VI	20 μg/L Penoxsulam + 50 mg/L BBE

### Morphological parameters

About 50 bulbs (each group) were chosen for the determination of root length using a millimeter ruler. The weight gain was evaluated before and after treatment using precision scales. The relative injury rate and rooting percentage were calculated using Eqs. 1 and 2^40^.

1 $$\text{Relative injury rate}=\frac{\%\text{Rooted bulbs in control}- \%\text{Rooted bulbs in each group}}{\%\text{Rooted bulbs in control}}$$

2 $$\text{Percentage of rooting}\left(\text{\%}\right)=\frac{\text{Rooted Bulbs}}{\text{Total Number of Bulbs}}$$

### Determination of Mitotic Index (MI), Chromosomal Aberrations (CAs) and Micronucleus (MN)

The root tips were fixed in ethanol: glacial acetic acid (3:1) for 2 h followed by 96% ethanol for 15 min. Then samples were processed in 70% ethanol at 4 °C. After hydrolysis in 1N HCl at 60 °C for 17 min, root tips were incubated with acetic acid (45%) for 30 min and stained in acetocarmine for 24 h. The mitotic index was calculated by counting cells in different mitotic phases and the total number of cells^41^. The assessment for the MN has been carried out according to Fenech et al*.*^42^.

About 10 slides per group were prepared from randomly selected bulbs, 1000 cells for MN and CAs, and 10,000 cells for MI were counted in each slide.

### Comet assay

The assay was performed according to the modified method of Tice et al*.*^43^. The roots were kept in petri dishes placed on ice. The nuclei were isolated in 600 µL ice-cold nuclear isolation buffer (400 mM 6H_2_O-MgCl_2_, 0.5% w/v Triton X-100, 0.4 M Tris, pH 7.5) using the razor blade. A mixture of low melting point agarose in phosphate-buffered saline (1%) and nuclear suspension in a ratio of 1:1 was placed on slides precoated with 1% normal melting point agarose. Electrophoresis was performed in a horizontal gel electrophoresis tank having chilled electrophoresis buffer at 0.7 V/cm (20 V, 300 mA) for 15 min at 4 °C. Then slides were rinsed with distilled water and neutralized in Tris buffer (0.4 M Tris, pH 7.5). The nuclei were stained with ethidium bromide after immersion in cold water. The slides were washed with cold water to eliminate the residual stain and sealed with coverslips. The procedure was followed in low light to avoid DNA degradation and slides were examined under a fluorescence microscope. Comets were analyzed using Comet Assay software version 1.2.3b^44^. About 100 cells per slide were analyzed for DNA damage. The extent of DNA damage was scored from 0 to 4 depending upon the level of DNA damage. The degree of DNA damage was graded on a scale of 0 to 4 based on the severity of DNA damage. The cells were divided into five groups depending on the length of their tail DNA, which ranged from zero to four^45^. The total DNA damage per sample, expressed as arbitrary units, was calculated using Eq. 3.

3 $$Arbitrary \,unit= \sum_{i=0}^{4}Ni x i$$

(*i*: degree of damage (0, 1, 2, 3, 4), *Ni:* the number of cells in *i* degree).

### Evaluation of antimutagenic effects

The antimutagenic effects were assessed using the arbitrary unit of comet assay and chromosomal aberrations (CAs) in Eq. 4^18^.

4 $$\text{Mutagenicity inhibition }\left({\%}\right)=\frac{\text{Penoxsulam Group Damage }\left({\%}\right)-\text{Penoxsulam with BBE Group Damage }\left({\%}\right)}{\text{Penoxsulam Group Damage }\left({\%}\right)-\text{ Control Group Damage }\left(\text{\%}\right)}\times 100$$

### Lipid peroxidation

Lipid peroxidation was evaluated according to the modified method of Ünyayar et al.^46^ by measuring the quantity of MDA. About 0.5 g root were homogenized in 5% trichloroacetic acid (TCA) and centrifuged at 12,000 rpm for 15 min at 24 °C. The supernatant was transferred to a new test tube having 20% TCA solution and 0.5% thiobarbituric acid and incubated for 25 min at 96 °C. The tubes were placed into the ice bath and centrifuged at 10,000 rpm for 5 min. The absorbance was recorded at 532 nm.

### Antioxidant enzyme assays

Superoxide dismutase (SOD) [EC 1.15.1.1] activity was determined according to the modified method of Beauchamp and Fridovich^47^. About 0.5 g root was homogenized in 5 mL of chilled sodium phosphate buffer (50 mM, pH 7.8). The homogenates were centrifuged at 10,500 rpm for 20 min. The 3 mL reaction mixture consisted 1.5 mL of sodium phosphate buffer (0.05 M, pH 7.8), 0.3 mL of methionine (130 mM), 0.3 mL of disodium EDTA (0.1 mM), 0.3 mL of nitroblue tetrazolium chloride (750 µM), 0.3 mL of riboflavin (20 μM), 0.01 mL of insoluble polyvinylpyrrolidone (4% w/v), 0.01 mL of enzyme extract and 0.28 mL of deionized water. The reaction started with keeping test tubes under two 15 W fluorescent lamps for 10 min and stopped by keeping the tubes in the dark for 10 min. Absorbance was measured at 560 nm. One unit of SOD enzyme activity was expressed as the amount of SOD enzyme required for 50% inhibition of NBT.

Catalase (CAT) [EC 1.11.1.6] level was assessed by the method of Beers and Sizer^48^ with alterations. The reaction mixture containing 0.3 mL of H_2_O_2_ (0.1 M), 1.5 mL of sodium phosphate (200 mM) and 1 mL of deionized water was formulated. The reaction was triggered by adding 0.2 mL of enzyme extract. CAT activity was measured by monitoring the decrease in absorbance decrease at 240 nm. CAT activity was determined as units per minute per g fresh weight.

Glutathione reductase (GR) [EC 1.8.1.7] activity was estimated using the modified method of Carlberg and Mannervik^49^. The root tips (0.5 g) were homogenized in the solution of EDTA (0.2 M, pH 4.7). The 2 mL reaction mixture containing 0.1 mM nicotinamide adenine dinucleotide phosphate (NADPH), 1 M oxidized glutathione (GSSG), 3 mM EDTA and 0.05 M potassium phosphate buffer (pH 7.0). The absorbance was recorded at 340 nm. GR activity was expressed as μmol NADPH/min.g FW.

### Molecular docking

The interactions of penoxsulam with antioxidant enzymes (GR, CAT and SOD) and DNA were analyzed by molecular docking. The 3D (crystallographic) structures of DNA (PDB ID: 1cp8)^50^, B-DNA dodecamer d (PDB ID: 195d)^51^, B-DNA dodecamer (PDB ID: 1bna)^52^, GR (PDB ID: 2hqm)^53^, CAT (PDB ID: 5gkn)^54^ and SOD (PDB ID: 1ba9)^55^ were retrieved from protein data bank. Penoxsulam 3D structure was obtained from PubChem. Biovia Discovery Studio 2020 Client was used for docking as well as analysis and 3D structure visualization. The active sites of enzymes were ascertained by removing co-crystal ligands and water molecules. However, polar hydrogen was added to enzymes. Gromos 43B1 using Swiss-Pdb Viewer (v.4.1.0) software was used for the minimization of enzymes energy^56^. Further, Open Babel v.2.4.0 software was employed for energy minimization of penoxsulam 3D structure^57^. The molecular docking procedure was initiated with DNA structure and grid box having active sites of enzymes. Autodock 4.2.6 software on was applied for docking. Lamarckian genetic algorithm was run for 10 times with a population size of 150 individuals for both DNA and enzymes.

### Dose–response effects of BBE

The dose–response effects of blueberry extract (BBE) against penoxsulam were calculated as the restorative effect of blueberry extract against penoxsulam toxicity on all parameters. The recovery percentage influenced by BBE was calculated by using proportion with the control group and penoxsulam application group data. Equation 5 was employed and evaluated with log values of doses^58^.

5 $$\text{Recovery Effect of BBE }\left(\text{\%}\right)=\frac{\text{Penoxsulam with BBE Group Parameter}-\text{Penoxsulam Group Parameter}}{\text{Control Group Parameter}-\text{ Penoxsulam Group Parameter}}\times 100$$

### Data analysis

Root elongation kinetics were analyzed using different mathematical models such as logistic, log-logistic, Gompertz, and Weibull. The selection of the most suitable model was determined based on their fit to Bayesian Information Criterion (BIC) and Akaike Information Criterion (AIC). The maximum growth (A), growth rate (µ), and length of lag phase (λ) were the common parameters used by these models to describe the growth patterns. The analysis was performed using the "tidyverse" and "drc" packages in the R programming language^59^. The analysis of correlations, and heat map of mitotic index were conducted using the Rstudio version 1.4.1106 software^60^. For the Pearson correlation analysis (two-tailed) and visualization, the Hmisc and corrplot package^61^ were utilized.

The statistical analysis was carried out using the SPSS Statistics v22.0 software package (IBM Corp., USA, 2015). The data were presented as mean ± SD (standard deviation) in tables and mean ± SEM (standard error of means) in graphs. One-way ANOVA and Duncan’s test were used to determine the statistical significance between means, with p < 0.05 considered statistically significant. For the analysis of mutagenicity inhibitions, independent samples t-test was used due to the presence of two independent variables, with p < 0.05 considered significant.

## Results

### Effect on root growth

The effects of penoxsulam and the protective role of blueberry extract were assessed by root length, rooting percentage, relative injury and weight gain (Table 2). The highest rooting percentage (100%) was recorded in group II (25 mg/L blueberry extract) and group III (50 mg/L blueberry extract) followed by group I (control) i.e. 96%. The application of 20 µg/L penoxsulam in group IV significantly decreased the rooting percentage (34%). However, the combined application of penoxsulam and blueberry extract in group V (20 µg/L penoxsulam + 25 mg/L blueberry extract) and group VI (20 µg/L penoxsulam + 50 mg/L blueberry extract) improved rooting percentage to 42% and 54% respectively.

**Table 2:** Effects of penoxsulam and BBE on morphological parameters.

Groups	Rooting percentage (%)	Mean root length	Weight gain (g)	Relative Injury Rate
Group I	96	10.15 ± 1.20^b^	8.16 ± 2.27^a^	–
Group II	100	10.64 ± 1.34^ab^	8.82 ± 1.08^a^	–
Group III	100	10.88 ± 1.37^a^	8.88 ± 1.17^a^	–
Group IV	34.00	5.10 ± 1.03^d^	2.90 ± 1.13^d^	0.65
Group V	42.00	6.89 ± 1.17^c^	4.25 ± 0.44^c^	0.56
Group VI	54.00	7.52 ± 1.40^b^	5.69 ± 0.79^b^	0.44

Group I: tap water (control), Group II: 25 mg/L BBE, Group III: 50 mg/L BBE, Group IV: 20 μg/L penoxsulam, Group V: 20 μg/L penoxsulam + 25 mg/L BBE, Group VI: 20 μg/L penoxsulam + 50 mg/L BBE. Data are shown as mean ± standard deviation (SD). The averages shown with different letters in the same column are statistically significant (P < 0.05).

The toxicity of penoxsulam is represented by impairing root length. The mean root length in group I, group II and group III was 10.15, 10.64 and 10.88 cm respectively. The application of 20 µg/L penoxsulam in group IV decreased root length (5.10 cm) by 50.5%. However, the application of 25 mg/L (group V) and 50 mg/L (group VI) blueberry extracts with penoxsulam increased root length to 6.89 and 7.52 cm respectively.

The effect of penoxsulam and blueberry extracts on weight gain was also recorded. The highest weight gain was observed in group III as 8.88 g, followed by group II as 8.82 g and group I as 8.16 g. The application of penoxsulam decreased weight gain to 2.90 g whereas treatment of blueberry extracts recovered weight gain in group V as 4.25 g and group VI as 5.69 g.

The relative injury rate indicates the intensity of the damage. The highest injury rate (0.65) was observed in penoxsulam-treated roots in group IV whereas the application of blueberry extracts decreased the injury rate in a concentration-dependent manner in group V and group VI to 0.56 and 0.44 respectively.

**Figure 1:**
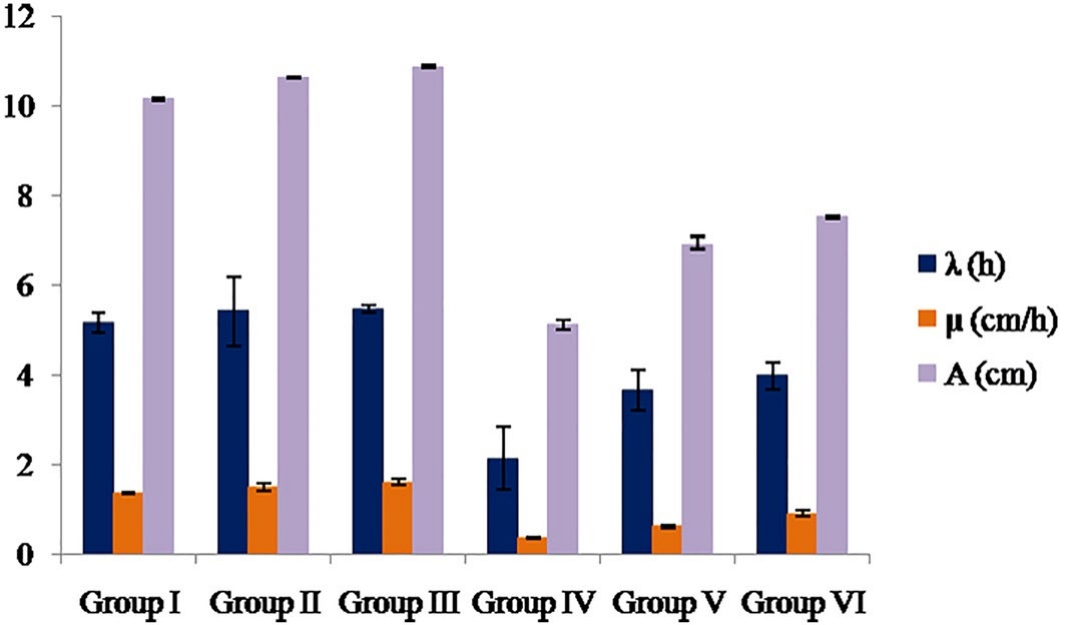
Effects of penoxsulam and blueberry treatments on root elongation kinetics. (Group I: tap water (control), Group II: 25 mg/L BBE, Group III: 50 mg/L BBE, Group IV: 20 μg/L penoxsulam, Group V: 20 μg/L penoxsulam + 25 mg/L BBE, Group VI: 20 μg/L penoxsulam + 50 mg/L BBE. Data are shown as mean ± standard deviation (SD).

The parameters of root elongation kinetics are sensitive endpoints related to pesticide toxicity. The application of blueberry extracts (25 and 50 mg/L) increased root length (A), growth rate (µ) and lag phase (λ) in group II and group III in comparison to the control. However a significant reduction in A, µ and λ was recorded in penoxsulam-treated roots (group IV). The treatment of blueberry extracts (25 and 50 mg/L) with penoxsulam in group V and group VI improved all the parameters of root elongation kinetics (Fig. 1).

### Effect on mitotic activity and chromosomal aberrations

**Figure 2:**
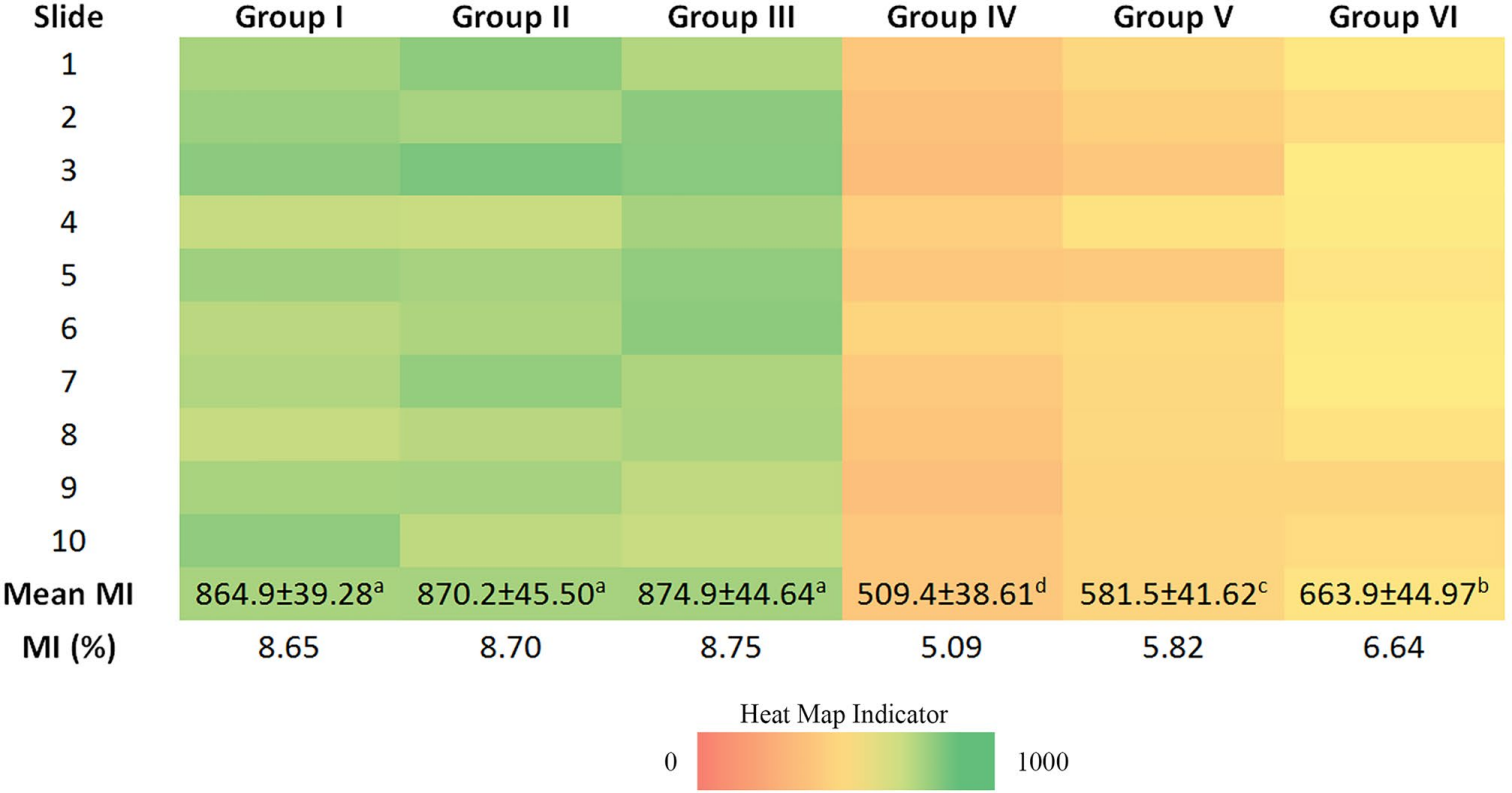
Effects of penoxsulam and BBE on mitotic index. [Group I: tap water (control), Group II: 25 mg/L BBE, Group III: 50 mg/L BBE, Group IV: 20 μg/L penoxsulam, Group V: 20 μg/L penoxsulam + 25 mg/L BBE, Group VI: 20 μg/L penoxsulam + 50 mg/L BBE. Data are shown as mean ± standard deviation (SD). The averages shown with different letters in the same line are statistically significant (P < 0.05)].

Formation of micronucleus (MN), chromosomal aberrations (CAs) and mitotic index (MI) were examined to study the cytogenetic effects of penoxsulam and blueberry extracts. The results revealed no significant difference in MI of group I (8.65%), group II (8.70%) and group III (8.75%). Treatment of penoxsulam decreased MI (5.09%) in group IV. However application of blueberry extracts (25 and 50 mg/L) with penoxsulam recovered MI in a concentration-dependent manner in group V (5.82%) and group VI (6.64%) indicating protective effects of blueberry extract (Fig. 2).

**Figure 3:**
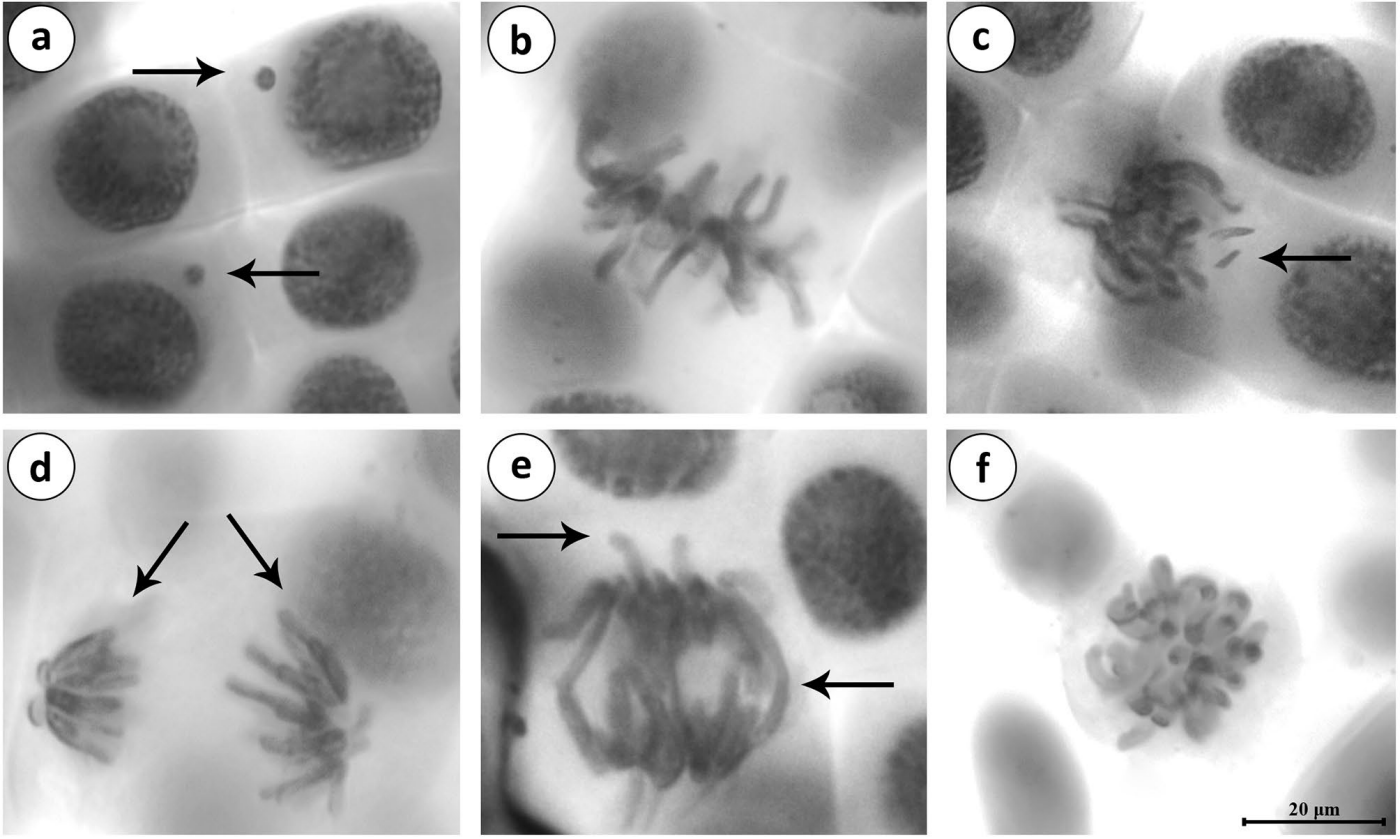
CAs and MN formations induced by penoxsulam (**a** MN, **b** sticky chromosome, **c** fragment, **d** unequal distribution of chromatin, **e** bridge and vagrant chromosome, **f** c-mitosis. Scale bar = 20 μm).

Various types of CAs (Fig. 3) such as sticky chromosome, bridge, fragment, vagrant chromosome, unequal distribution of chromatin, c-mitosis and micronucleus formations were observed in this study (Tables 3, 4). Few abnormal cells with sticky chromosomes and micronucleus were reported in group I and group II. No CAs were observed in group II. There was no significant difference in CAs noted in group I, group II and group III. The high frequency of various aberrations was observed in the roots treated with penoxsulam (30.63%). The application of blueberry extracts at 25 and 50 mg/L recovered CAs induced by penoxsulam in group V (23.17%) and group VI (17.25%) indicating the antimutagenic effect of blueberry extract.

**Table 3:** Effects of penoxsulam on MN formation and protective role of BBE.

Groups	Minimum MN	Maximum MN	Mean MN Formation
Group I	0.00	1.00	0.40 ± 0.52^d^
Group II	0.00	1.00	0.20 ± 0.42^d^
Group III	0.00	0.00	0.00 ± 0.00^d^
Group IV	36.00	53.00	44.70 ± 6.80^a^
Group V	22.00	40.00	32.20 ± 5.55^b^
Group VI	12.00	30.00	19.80 ± 5.22^c^

Group I: tap water (control), Group II: 25 mg/L BBE, Group III: 50 mg/L BBE, Group IV: 20 μg/L penoxsulam, Group V: 20 μg/L penoxsulam + 25 mg/L BBE, Group VI: 20 μg/L penoxsulam + 50 mg/L BBE. Data are shown as mean ± standard deviation (SD). The averages shown with different letters in the same column are statistically significant (P < 0.05).

**Table 4:** Frequencies of chromosomal aberrations (CAs) induced by penoxsulam and the protective role of BBE.

	Group I	Group II	Group II	Group IV	Group V	Group VI
SC	0.60 ± 0.84	0.10 ± 0.32	0.00 ± 0.00	73.70 ± 8.72	57.60 ± 5.15	44.30 ± 6.36
FRG	0.10 ± 0.32	0.00 ± 0.00	0.00 ± 0.00	69.10 ± 5.92	55.90 ± 3.93	43.90 ± 6.06
UDC	0.00 ± 0.00	0.00 ± 0.00	0.00 ± 0.00	58.30 ± 5.31	43.50 ± 4.50	29.30 ± 4.88
B	0.00 ± 0.00	0.00 ± 0.00	0.00 ± 0.00	44.90 ± 8.28	31.30 ± 6.27	20.70 ± 3.95
VC	0.00 ± 0.00	0.00 ± 0.00	0.00 ± 0.00	34.50 ± 3.63	25.30 ± 5.77	18.90 ± 4.20
CM	0.00 ± 0.00	0.00 ± 0.00	0.00 ± 0.00	25.80 ± 3.94	18.10 ± 4.04	15.40 ± 3.75
Total CAs	0.70 ± 0.82^d^	0.10 ± 0.32^d^	0.00 ± 0.00^d^	306.30 ± 32.88^a^	231.70 ± 26.51^b^	172.50 ± 17.04^c^
CAs (%)	0.07	0.01	0	30.63 ± 3.29^a^	23.17 ± 2.65^b^	17.25 ± 1.70^c^
Mutagenicity inhibition(%)	–	–	–	–	24.41 ± 5.10^b^	43.78 ± 8.30^a^

Group I: tap water (control), Group II: 25 mg/L BBE, Group III: 50 mg/L BBE, Group IV: 20 μg/L penoxsulam, Group V: 20 μg/L penoxsulam + 25 mg/L BBE, Group VI: 20 μg/L penoxsulam + 50 mg/L BBE. Data are shown as mean ± standard deviation (SD). The averages shown with different letters in the same column are statistically significant (P < 0.05). SC: Sticky chromosome, FRG: Fragment, UDC: Unequal distribution of chromatin, B: Bridge, VC: vagrant chromosome CM: c-mitosis.

**Figure 4:**
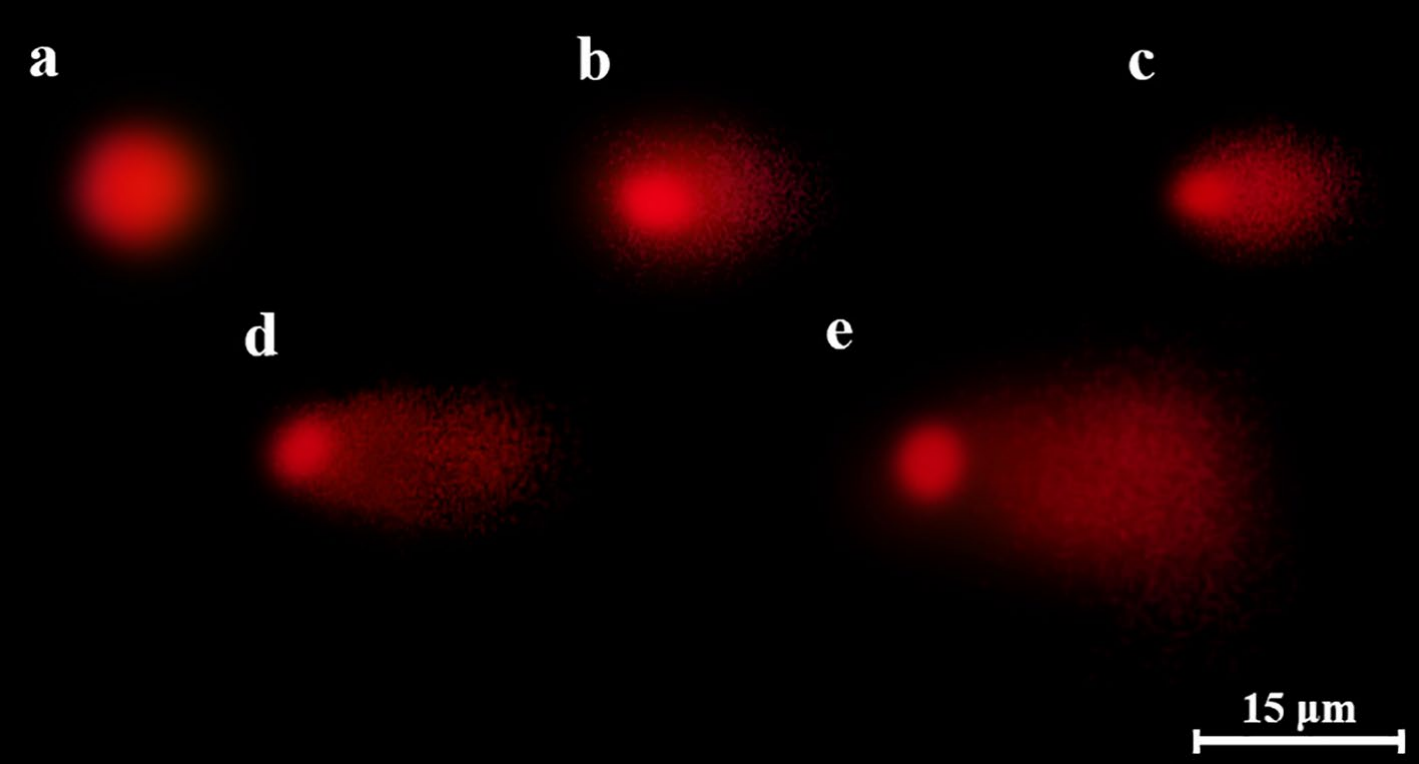
Effects of penoxsulamon DNA in *A. cepa* root tip cells (**a** no damage, **b** low damage, **c** moderate damage, **d** high damage, **e** extreme damage. Scale bar = 15 μm).

The genotoxic effect of penoxsulam and the protective role of the blueberry extract was evaluated using single-cell gel electrophoresis (SCGE) with three parameters including tail DNA, head DNA and arbitrary unit. Figure 4a shows the nuclei of undamaged cells in the control group, while Fig. 4b–e represents various degrees of DNA damage observed in different treated groups. The results showed no significant difference in tail DNA (%), head DNA (%) and arbitrary unit among group I, group II and group III. However, a significant difference in all the parameters of SCGE was observed among group IV, group V and group VI. Penoxsulam treatment increased tail DNA to 60.43%, arbitrary unit to 570.10 whereas decreased head DNA to 39.57%. In addition, the application of different doses of blueberry extracts with penoxsulam decreased tail DNA% and arbitrary unit while increasing head DNA%.

Moreover, the mutagenicity inhibition (Table 5) was calculated using DNA damage in group I (control), group IV (penoxsulam treatment), group V and group VI (penoxsulam + blueberry extract). The results indicated a concentration-dependent mutagenicity inhibition by blueberry extracts. The administration of 25 mg/L and 50 mg/L blueberry extracts with penoxsulam inhibited mutagenicity by 28.38% and 37.90% respectively.

**Table 5:** Detection of the effects of penoxsulam and BBE applications on DNA by comet assay.

Groups	Head DNA (%)	Tail DNA (%)	Arbitrary unit	Mutagenicity inhibition (%)
Group I	98.37 ± 1.42^a^	1.63 ± 1.42^d^	40.50 ± 4.67^d^	–
Group II	98.44 ± 1.35^a^	1.56 ± 1.35^d^	38.60 ± 6.13^d^	–
Group III	98.46 ± 1.34^a^	1.54 ± 1.34^d^	36.10 ± 6.77^d^	–
Group IV	39.57 ± 22.74^d^	60.43 ± 22.74^a^	570.10 ± 28.05^a^	–
Group V	58.89 ± 22.52^c^	41.11 ± 22.52^b^	419.80 ± 36.83^b^	28.38 ± 3.20^a^
Group VI	65.23 ± 20.50^b^	34.77 ± 20.50^c^	369.40 ± 29.17^c^	37.90 ± 5.10^a^

Group I: tap water (control), Group II: 25 mg/L BBE, Group III: 50 mg/L BBE, Group IV: 20 μg/L penoxsulam, Group V: 20 μg/L penoxsulam + 25 mg/L BBE, Group VI: 20 μg/L penoxsulam + 50 mg/L BBE. Data are shown as mean ± standard deviation (SD). The averages shown with different letters in the same column are statistically significant (P < 0.05).

### Effect on lipid peroxidation and antioxidant enzyme activities

**Figure 5:**
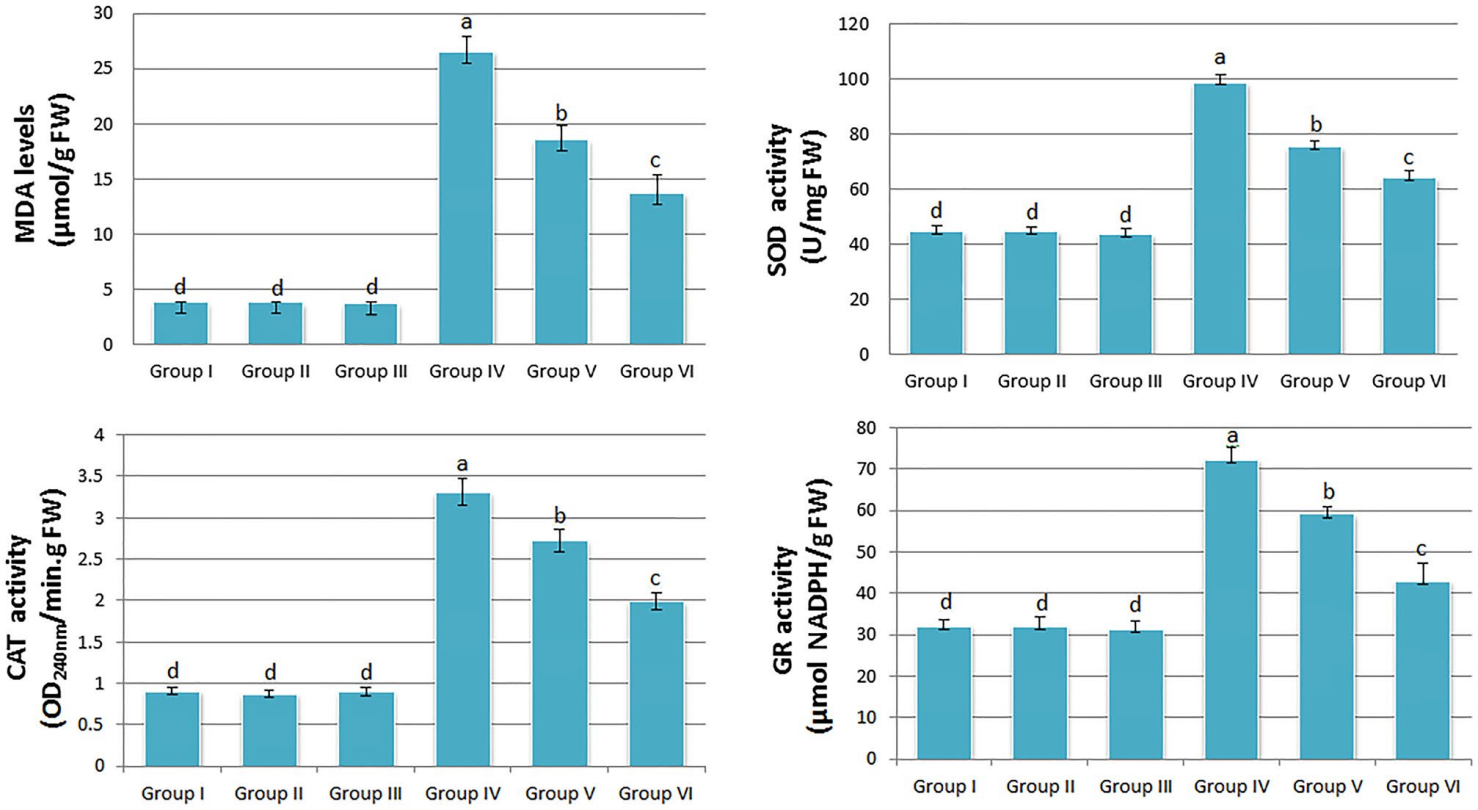
Effects of penoxsulam and BBE applications on lipid peroxidation and antioxidant enzyme activities (**a:** MDA levels, **b:** SOD activity, **c:** CAT activity** d**: GR activity) [Group I: tap water (control), Group II: 25 mg/L BBE, Group III: 50 mg/L BBE, Group IV: 20 μg/L penoxsulam, Group V: 20 μg/L penoxsulam + 25 mg/L BBE, Group VI: 20 μg/L penoxsulam + 50 mg/L BBE. Data were shown as mean ± SEM. The averages shown with different letters in each graph are statistically significant (P < 0.05)].

Malondialdehyde (MDA) content is a marker to assess lipid peroxidation in the membrane. Lipid peroxidation is considered the first step of membrane damage. The results showed no significant difference in MDA content in group I (3.83 µmol/g FW), group II (3.80 µmol/g FW) and group III (3.76 µmol/g FW). Penoxsulam treatment significantly increased the MDA level (26.54 µmol/g FW) in comparison to the control. The application of different doses of blueberry extracts with penoxsulam in group V and group VI significantly recovered MDA content by 30.11% and 48.46% respectively (Fig. 5a).

The antioxidant defense system alleviates and repairs ROS damage in plants. In the present study, the increased activities of antioxidant enzymes (SOD, CAT and GR) after penoxsulam treatment indicated the activation of the ROS scavenging mechanism. SOD activity was influenced by the level of superoxide radicals in plants. SOD activity in group I, group II and group III was insignificant and recorded as 44.67, 44.88 and 43.80 U/mg FW respectively. SOD activity in group IV, group V and group VI was significantly different and noted as 98.85, 75.67 and 64.38 U/mg FW (Fig. 5b).

CAT scavenges the harmful effects of hydrogen peroxide by converting it into water and oxygen. CAT activity was insignificant in group I (0.91 OD_240nm_/min.g FW), group II (0.87 OD_240nm_/min.g FW) and group III (0.90 OD_240nm_/min.g FW). However, a significant rise in CAT activity (3.31 OD_240nm_/min.g FW) was noted in the penoxsulam-treated group. Further, administration of blueberry extract with penoxsulam decreased CAT activity in group V (2.73 OD_240nm_/min.g FW) and group VI (1.99 OD_240nm_/min.g FW) (Fig. 5c).

GR manages the concentration of reduced glutathione in the cell. The reduced glutathione plays a key role in ROS homeostasis. The result showed insignificant GR activity in group I (32.21 µmol NADPH/g FW), group II (32.19 µmol NADPH/g FW) and group III (31.42 µmol NADPH/g FW). GR activity (72.50 µmol NADPH/g FW) was significantly high in the penoxsulam-treated group in comparison to other groups. However, administration of blueberry extracts showed a concentration-dependent reduction in GR activity in group V (59.22 µmol NADPH/g FW) and group VI (43.17 µmol NADPH/g FW) (Fig. 5d).

**Figure 6:**
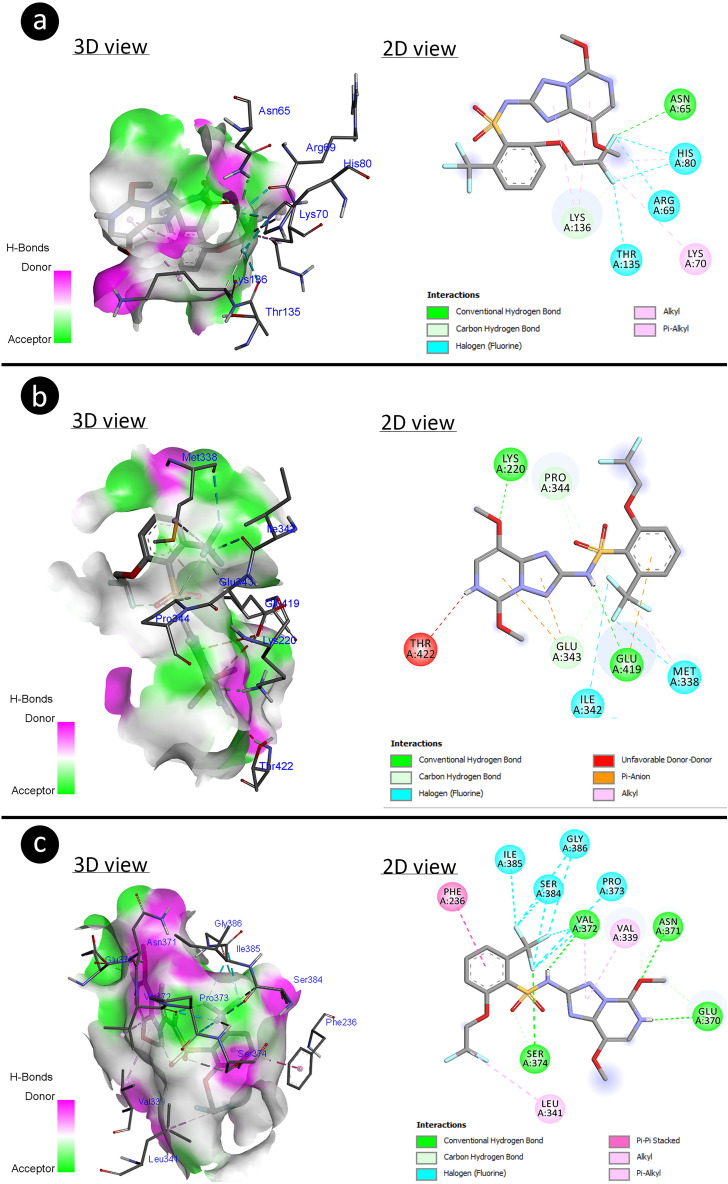
Molecular insights of penoxsulam interaction with antioxidant enzyme residues (**a** SOD, **b** CAT, **c** GR).

Figure 6 and Table 6 explain the interactive potential of penoxsulam with different antioxidant enzymes (SOD, CAT and GR). The penoxsulam made H-bond interaction to residues (ASN65 and LYS136), halogen interactions (ASN65, LYS136, ARG69, HIS80 (× 2), and THR135) and hydrophobic interactions (LYS70, HIS80, and LYS136 (× 2)) of SOD with binding energy (− 2.21 kcal/mol) and inhibition constant (Ki: 24.10 mM). The residues (LYS220, GLU419, GLU343, and PRO344 (× 2)) of CAT have interacted with penoxsulam through H-bond and other halogenation (GLU343, MET338, and ILE342) and hydrophobic (MET338) interaction were accomplished with binding energy (− 2.90 kcal/mol) and inhibition constant (7.44 mM). The H-bond (ASN371, SER374, GLU370 (× 2), VAL372, and SER374), halogenation (SER374, VAL372 (× 2), PRO373, SER384 (× 2), ILE385, and GLY386 (× 2)) and hydrophobic (PHE236, LEU341, VAL339, and VAL372) interaction were participated in making complexation with GR by involving binding energy (− 5.66 kcal/mol) and inhibition constant (70.39 μM).

**Table 6:** Molecular interactions of Penoxsulam with antioxidant enzymes.

Antioxidant enzymes	Free energy of binding (kcal/mol)	Inhibition constant (Ki)	Hydrogen bond interactions	Halogen interactions	Hydrophobic interactions
SOD	− 2.21	24.10 mM	ASN65 LYS136	ASN65 LYS136 ARG69 HIS80 (× 2) THR135	LYS70 HIS80 LYS136 (× 2)
CAT	− 2.90	7.44 mM	LYS220 GLU419 GLU343 PRO344 (× 2)	GLU343 MET338 ILE342	MET338
GR	− 5.66	70.39 μM	ASN371 SER374 GLU370 (× 2) VAL372 SER374	SER374 VAL372 (× 2) PRO373 SER384 (× 2) ILE385 GLY386 (× 2)	PHE236 LEU341 VAL339 VAL372

*SOD* Superoxide dismutase, *CAT* Catalase and *GR*: Glutathione reductase. Aminoacids; *ARG* Arginine, *ASN* Asparagine, *GLU* Glutamic acid, *GLY* Glycine, *HIS* Histidine, *ILE* Isoleucine, *LYS* Lysine, *MET* Methionine, *PHE* Phenylalanine, *PRO* Proline, *SER* Serine, *THR* Threonine, *VAL* Valine.

**Figure 7:**
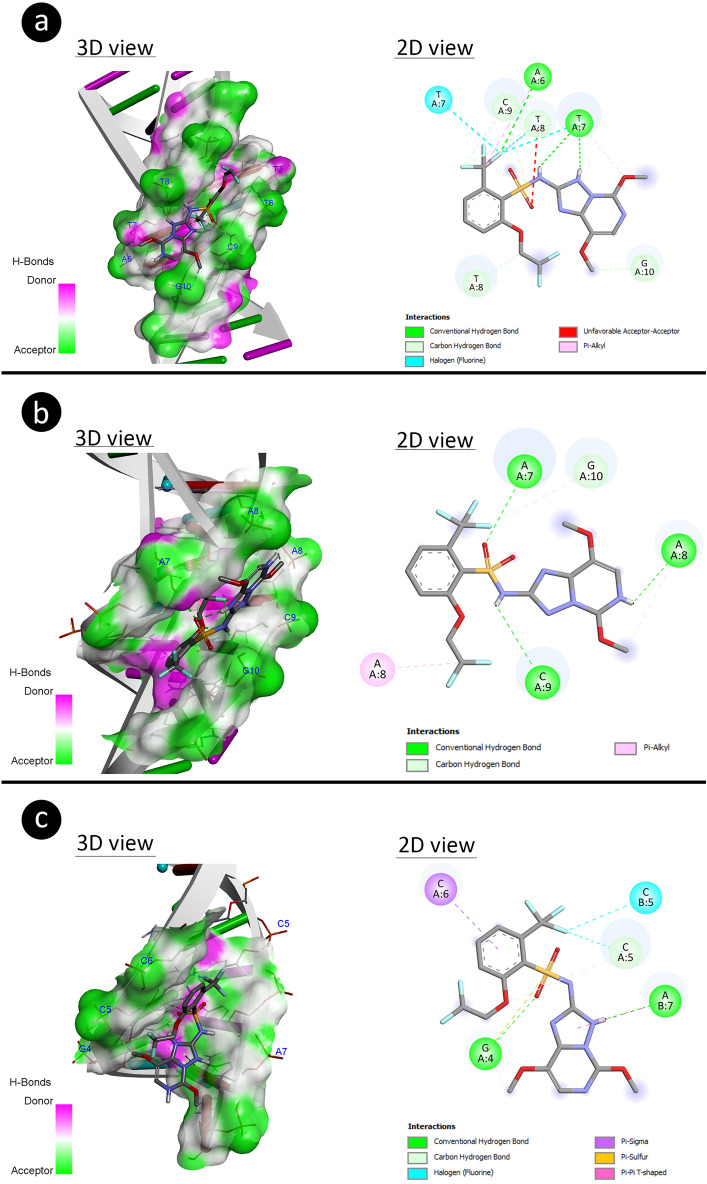
The structural interaction of penoxsulam with DNA (**a **1BNA, **b **195D, **c **1CP8).

The B-DNA Dodecamer (1BNA) had made a complex with penoxsulam through its bases (A6, T7, T8, C9, and G10) of nucleic acid interaction using binding energy (− 6.73 kcal) and inhibition constant (11.58 μM). The penoxsulam has integrated with bases (A7, A8, C9, and G10) of B-DNA Dodecamer D (195D) dissipating binding energy (− 5.22 kcal/mol) with inhibition constant (Ki: 149.19 μM). The bases (G4, C5, C6, and A7) of DNA (1CP8) have complexed with penoxsulam by involving binding energy (-5.21 kcal/mol) with inhibition constant (Ki:152.02 μM). The above explanation about the interactive efficiencies of penoxsulam toward DNA has been mentioned in Fig. 7 and Table 7.

**Table 7:** The binding energy and molecular interactions of the penoxsulam with DNA molecules.

DNA Molecule	DNA Sequence	Free energy of binding (kcal/mol)	Inhibition constant (Ki)	Interacting nucleic acids
B-DNA Dodecamer (1BNA)	5′-CGCGAATTCGCG-3′	− 6.73	11.58 μM	A6 T7 T8 C9 G10
B-DNA Dodecamer D (195D)	5′-CGCGTTAACGCG-3′	− 5.22	149.19 μM	A7 A8 C9 G10
DNA (1CP8)	5′-TTGGCCAA-3′	− 5.21	152.02 μM	G4 C5 C6 A7

Nucleic acids; *A* Adenine, *C* Cytosine, *G* Guanine, *T* Thymine.

### Concentration-dependent protecting effects of blueberry extracts and their correlations

**Figure 8:**
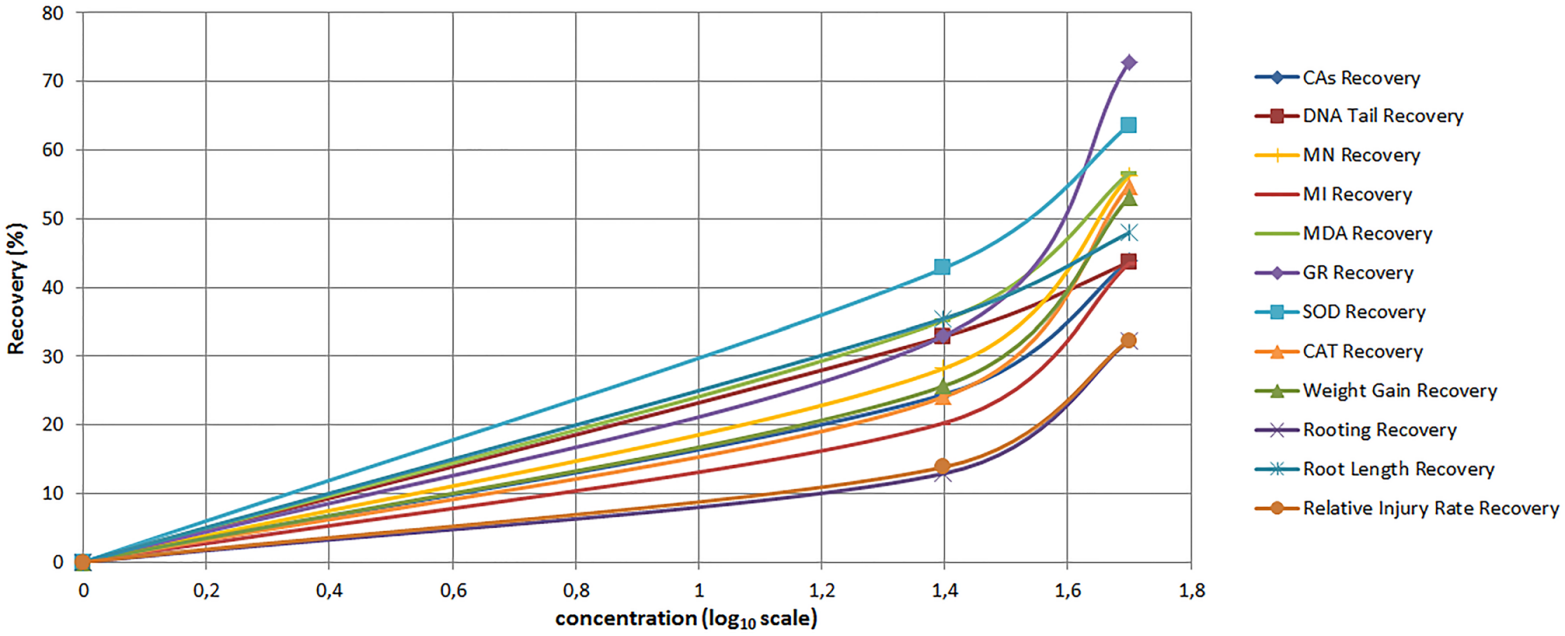
Dose–response protection curves of BBE against penoxsulam toxicity.

Figure 8 shows the protective effects of blueberry extracts in a dose-dependent manner against penoxsulam toxicity. The dose–response curve indicated improvement in all the parameters after the application of blueberry extracts. The highest protective effect was observed at 50 mg/L concentration of blueberry extract. This dose of blueberry extract improved genetic parameters from 43.64 to 56.21%, morphological parameters from 32.26 to 53.04% and antioxidant enzymes with lipid peroxidation from 54.77 to 72.80%. In addition, GR activity had shown the highest recovery of 72.80%. The results also suggested a direct or indirect linkage among all the parameters.

**Figure 9:**
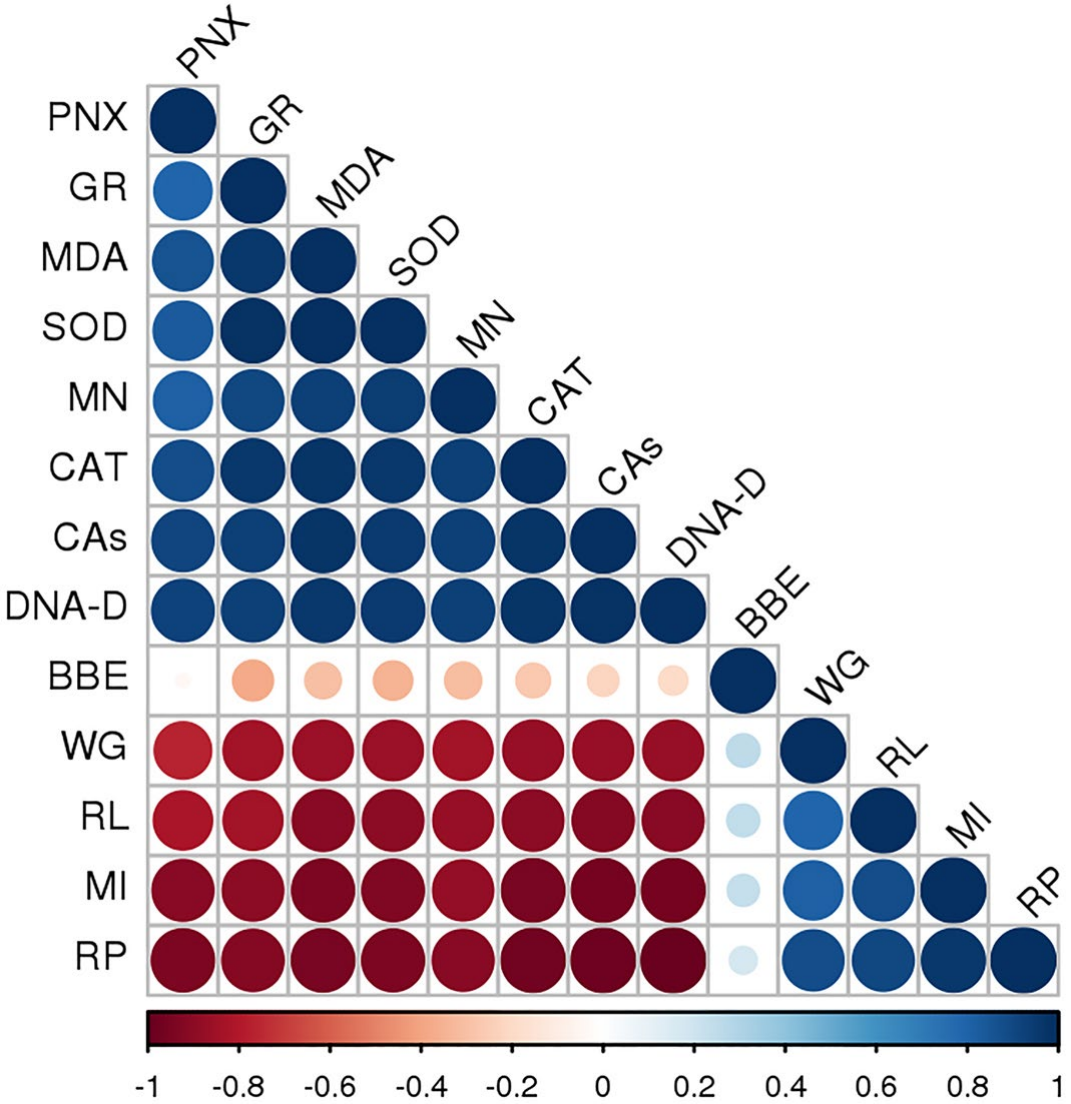
Correlations of penoxsulam and BBE doses with cytological, morphologic and biochemical analysis (*PXM* penoxsulam dose, *GR* glutathione reductase activity, *MDA* malondialdehyde level, *SOD* superoxide dismutase activity, *MN* micronucleus formation, *CAT* catalase activity, *CAs* chromosomal abnormality frequency, *DNA-D* DNA damage (arbitrary unit), *BBE* blue berry extract, *WG* weight gain, *RL* root length, *MI* mitotic index, *RP* rooting percentage).

The correlation study of penoxsulam and blueberry extracts with morphological parameters, genetic parameters and antioxidant enzyme levels with lipid peroxidation is shown in Fig. 9. The blue color indicates a positive correlation while red color specifies a negative correlation. The size of the circle and the intensity of color are indicators of correlation coefficients. A positive correlation was noted in penoxsulam with antioxidant enzyme activities (GR, SOD and CAT), lipid peroxidation, micronucleus, chromosomal aberrations and DNA damage (arbitrary unit) while negative correlations were observed for weight gain, root length, rooting percentage and mitotic index. However, blueberry extracts showed positive correlations with gain, root length, rooting percentage and mitotic index whereas negative correlations were recorded for other parameters indicating its protecting effects.

## Discussion

Herbicides like penoxsulam are commonly used to control weeds. They inhibit weed growth and protect crops from the nutrient competition. But their inappropriate use may lead to environmental pollution and hazardous effects on non-targeted organisms^62^. Penoxsulam influenced physiological parameters by decreasing rooting percentage, root length and weight gain whereas the application of blueberry extracts either alone or in combination with penoxsulam recovered these parameters. The kinetics of root elongation also suggested reductions in growth rate and root length in penoxsulam-treated *A. cepa* roots but blueberry extracts at 25 mg/L and 50 mg/L showed protecting effects by increasing growth rate and root length. The parameters of root elongation kinetics are sensitive endpoints to determine the phytotoxic effects of the herbicide. However, Ali et al*.*^62^ suggested that penoxsulam inhibits plant growth through a phytohormone-dependent pathway. The mitotic activity was also arrested after penoxsulam treatment indicating the cytotoxic effects of the herbicide. However, the application of blueberry extracts either alone or in combination with penoxsulam enhanced the mitotic index in a concentration-dependent manner emphasizing the protective role of blueberry extract. Similar inhibitory effects of penoxsulam on root growth and mitotic activity were reported by Özkan and Liman^63^. Panneerselvam et al*.*^64^ observed that recommended dose of penoxsulam significantly reduced the root growth and root colonization of arbuscular mycorrhizae fungi in rice.

Various types of CAs were determined in the present study. Penoxsulam treatment induced different types of CAs in the roots of *A. cepa* L. whereas application of blueberry extracts declined CAs in concentration-dependent mode. Sticky chromosome, a chromatid type aberration, may be result of DNA degradation/depolymerization by environmental pollutants. The presence of micronuclei, fragments and bridges demonstrates the clastogenic effects^65–67^. CAs like c-mitosis, vagrant chromosome and unequal distribution of chromatin may be originated due to the intervention of chemicals in spindle formation^68,69^. Kaya and Gül^70^ reported the antimutagenic and antigenotoxic effects of blueberry extract. These findings were confirmed by the studies of Fragiorge et al*.*^71^, Rad et al*.*^72^, Liman et al*.*^73^, Magdaleno et al*.*^74^, Soloneski et al*.*^75^ and Liman and Özkan^63^.

The comet assay is commonly used in environmental monitoring to evaluate the relationship between exposure to pollutants and DNA damage. DNA damage induced by pollutants may be the result of the direct interaction of environmental pollutants and their metabolites with DNA or due to oxidative stress^76^. The unrepaired damages cause DNA alterations. The results revealed that exposure of *A. cepa* roots to penoxsulam herbicide induced a high level of DNA damage indicating the genotoxic potential of herbicide. However, blueberry extracts reduced DNA damage at 25 mg/L and 50 mg/L demonstrating the concentration-dependent protective role of extract. Similar responses was reported in other organisms such as *Mytilus galloprovincialis*^14^ and *Procambarus clarkia*^77^. Szeto et al*.*^78^ suggested the genoprotective effect of blueberry on human lymphocytes against exogenous oxidative stress. Penoxsulam treatment may increase ROS production that can reduce mitotic activity, root growth and enhance CAs and DNA damage in *A. cepa* roots.

Earlier studies suggested that an imbalance between reactive oxygen species (ROS) production and ROS scavenging mechanism might have a negative impact on normal cell metabolism. The excess ROS may induce oxidative stress that causes protein oxidation, lipid peroxidation, DNA damage and aging effects^79^. The present study revealed a positive correlation among CAs, DNA damage, lipid peroxidation and antioxidant enzyme activities. Penoxsulam exposure enhanced antioxidant enzyme (SOD, CAT and GR) activities in the roots of *A. cepa* L. Although, the application of blueberry extracts with penoxsulam decreased antioxidant enzyme activities, the levels of SOD, CAT and GR was still higher in comparison to control. The decrease in antioxidant enzyme activities indicates protecting role of the blueberry extract by reducing ROS level in the cell^80^. The first line of defense against ROS is SOD which catalyzes superoxide radicals into oxygen and hydrogen peroxide. In the meantime, hydrogen peroxide is catalyzed by CAT. These reactions indicate the activation of antioxidant enzyme-dependent defense mechanisms against ROS. Moreover, GR maintains levels of reduced glutathione in the cell which plays important role in the detoxification of ROS. SOD, CAT and GR are important antioxidant enzymes for reducing oxidative stress^81,82^. Nohatto et al*.*^13^ reported that penoxsulam application influenced hydrogen peroxide level, lipid peroxidation and SOD enzyme activity in rice. However, Murussi et al*.*^3^ observed penoxsulam-induced oxidative stress in *Rhamdia* sp. and *Cyprinus carpio.*

Moreover, molecular docking showed the interactive capability of penoxsulam (3-D structure) to DNA and antioxidant enzymes. The results revealed higher binding energy (− 5.66 kcal/mol) for GR-complex indicating penoxsulam induced more perturbation in the 3-D structure of GR in comparison to CAT and SOD. The biochemical analysis results also suggested that increased antioxidant enzyme activities demonstrating more enzyme synthesis may lead to reduce oxidative stress. Penoxsulam interactions with the 3-D structure of DNA could alter DNA structure between adjacent DNA bases (1BNA: A6-T7-T8 and T7-T8-C9-G10, 195D: A7-A8 and A8-C9-G10, 1CP8: G4-C5-C6). The binding of penoxsulam between DNA bases may lead to intercalation reactions inducing genotoxicity^83,84^. Interaction with DNA can cause dissociation, chain breaks, disruption of its integrity, CAs and MN formations^85^.

The formation of MDA is an indicator of lipid peroxidation caused by ROS. The results showed a high level of lipid peroxidation in penoxsulam-treated roots indicating an imbalance between ROS production and scavenging activity that increased MDA level and oxidative stress. However, blueberry extracts reduced the severity of membrane damage when supplied with penoxsulam. Endogenous antioxidants protect cells from oxidative stress and lipid peroxidation^86^. Similarly, blueberry extracts showed its ROS scavenging properties decreasing oxidative stress in the human keratinocyte cell line^80^.

## Conclusion

In conclusion, penoxsulam exposure induced cytotoxicity, genotoxicity, oxidative stress and DNA damage in roots of *A. cepa* L. It also inhibited growth rate and root growth. Further, penoxsulam influenced normal cell function and enhanced MDA levels and antioxidant enzyme activities. The results of molecular docking suggested that penoxsulam could up-regulate antioxidant enzyme activities by combining with their residues. However, blueberry extract decreased the toxic effects of penoxsulam herbicide by reducing ROS levels that can activate oxidative reactions in cell membranes leading to lipid peroxidation. The end product of lipid peroxidation can cause carcinogenic and mutagenic effects forming DNA adducts by reacting with deoxyadenosine and deoxyguanosine. The ability of the blueberry extract to reduce DNA damage may be the result of diminished ROS level. Thus, the application of blueberry extract with the herbicide penoxsulam could minimize the negative effects on non-targeted organisms. As a result, it has been seen that the blueberry extract can tolerate all these toxic effects of penoxsulam depending on the concentration, and it has been understood that it is a good protective natural product against such chemical exposures.

## Data Availability

The datasets used and/or analysed during the current study available from the corresponding author on reasonable request.
